# Adolescent Changes in Dopamine D1 Receptor Expression in Orbitofrontal Cortex and Piriform Cortex Accompany an Associative Learning Deficit

**DOI:** 10.1371/journal.pone.0056191

**Published:** 2013-02-21

**Authors:** Anna K. Garske, Chloe R. Lawyer, Brittni M. Peterson, Kurt R. Illig

**Affiliations:** Department of Biology and Program in Neuroscience, University of St. Thomas, Saint Paul, Minnesota, United States of America; Université Lyon, France

## Abstract

The orbitofrontal cortex (OFC) and piriform cortex are involved in encoding the predictive value of olfactory stimuli in rats, and neural responses to olfactory stimuli in these areas change as associations are learned. This experience-dependent plasticity mirrors task-related changes previously observed in mesocortical dopamine neurons, which have been implicated in learning the predictive value of cues. Although forms of associative learning can be found at all ages, cortical dopamine projections do not mature until after postnatal day 35 in the rat. We hypothesized that these changes in dopamine circuitry during the juvenile and adolescent periods would result in age-dependent differences in learning the predictive value of environmental cues. Using an odor-guided associative learning task, we found that adolescent rats learn the association between an odor and a palatable reward significantly more slowly than either juvenile or adult rats. Further, adolescent rats displayed greater distractibility during the task than either juvenile or adult rats. Using real-time quantitative PCR and immunohistochemical methods, we observed that the behavioral deficit in adolescence coincides with a significant increase in D1 dopamine receptor expression compared to juvenile rats in both the OFC and piriform cortex. Further, we found that both the slower learning and increased distractibility exhibited in adolescence could be alleviated by experience with the association task as a juvenile, or by an acute administration of a low dose of either the dopamine D1 receptor agonist SKF-38393 or the D2 receptor antagonist eticlopride. These results suggest that dopaminergic modulation of cortical function may be important for learning the predictive value of environmental stimuli, and that developmental changes in cortical dopaminergic circuitry may underlie age-related differences in associative learning.

## Introduction

Learning to associate environmental cues with behaviorally significant outcomes is necessary for organizing and driving behavior. In rodents, this associative learning is thought to be mediated in large part by a highly interconnected neuronal network that includes the orbitofrontal cortex (OFC), amygdala, and piriform cortex [1]–[4]. In odor-guided behavioral tasks, cells in both the OFC and the piriform cortex can change their response to odors based on the incentive or reward value of the stimuli [4]–[8]. This shift in responding is mirrored by subcortical neurons; early in a behavioral task, midbrain dopamine neurons respond to the presentation of a reward, but after an association has been learned, responses are evoked by the predictive stimuli [9]–[13]. Cells in both the OFC and piriform cortex receive input from midbrain dopaminergic neurons, express dopamine receptors and exhibit dose-dependent responses to dopamine [14]–[21]. Further, dopamine modulation in the frontal cortex is hypothesized to be important in attending to external stimuli [22]–[23]. Therefore, dopaminergic input may be important for modulating response plasticity in cortical neurons, and thus for encoding predictive value in the OFC and piriform cortex; indeed, changes in the D1 receptor subtype have been implicated in response plasticity of frontal cortical regions [24]–[28].

In the rat, adult-like dopaminergic projections to the OFC are established between postnatal days 20–35 [29], and dopamine receptor expression in the brain changes substantially during this time [30]–[32]. We hypothesized that if dopamine input to OFC and piriform cortex is important in olfactory-guided associative learning, then any age-related changes in D1 receptor expression in these areas would affect learning on an olfactory-guided associative task. To investigate this question, we first tested juvenile, adolescent and adult animals on an olfactory-guided associative learning task. Next, we explored the postnatal development of D1 receptor mRNA and protein expression in the OFC and piriform cortex using quantitative real-time PCR and immunohistochemical methods. Finally, we used pharmacological manipulations to probe the involvement of dopamine in associative learning in adolescent rats.

## Materials and Methods

### Ethics Statement

All procedures in this study were conducted in accordance with guidelines for animal use published by the Society for Neuroscience and the National Institutes of Health, under protocol approved by the University of St. Thomas institutional animal care committee (Protocol #48).

### Subjects

We used 97 Male Long-Evans rats (Harlan Laboratories, Madison, WI) from four age groups: juvenile (21–28 days of age; approximate weight less than 90 g), adolescent (34–49 d; 110–199 g), adult (50–90 d; 200–450 g) and aged adult (>100 d; >500 g). Results from the two adult groups are presented together because there were no significant differences on any measures between these two groups.

### Experiment 1: Odor-guided Associative Learning Task

To compare the ability of juvenile, adolescent and adult animals to acquire an odor-based association, rats were trained on a digging task similar to that described previously (e.g., [33]–[35]). First, rats were trained to dig in a cup made from PVC pipe (10 cm diameter, 6 cm depth) filled with clean, unscented playground sand to obtain a palatable food reward (one quarter of a Froot Loop; Kellog’s). Initially, Froot Loop pieces were placed at the top of the sand. Once rats became acquainted with this arrangement, the pieces were buried progressively deeper into the sand on each trial, with trials continuing until Froot Loops were completely buried (>3 cm) and animals exhibited task mastery by digging for Froot Loops throughout an entire 20 minute session.

After training with unscented sand, animals were tested on their ability to perform an odor-association task, in which two sand-filled cups were presented to the rat, with each cup containing sand combined with a different odor (e.g., sand in cup 1 combined with cinnamon, sand in cup 2 combined with sage). Only one of the two odorized cups contained a Froot Loop. Digging in the Froot Loop-containing cup was considered a “correct” trial, while digging in the empty cup was considered “incorrect” and would result in a short timeout from the task. Sand was odorized using commercially-obtained fragrances, spices and extracts, diluted to a 10% concentration with mineral oil prior to combining with sand. Cups were randomly positioned within the arena for each trial, and no location was used more than three consecutive times. The task continued for a 20 minute session or until rats reached performance criterion (8 correct of 10 consecutive trials), whichever came first. The number of trials to achieve criterion was recorded for each rat; for rats that required more than one session, trials were counted across sessions. Those trials during which animals were inactive or displayed behavior that was not directed toward the sand-filled cups (e.g., grooming) for 30 consecutive seconds were counted as “distracted” trials, which were analyzed separately. Results from behavioral experiments were analyzed using one-way (e.g., trials to criterion X group) analyses of variance (ANOVA), and a significant F test was followed by Tukey’s HSD post-hoc test of group means as noted.

Rats from all age groups were trained and tested. Naïve rats began training at different ages, but were tested at all subsequent ages to examine effects of previous experience (e.g., naïve juvenile rats were tested as “experienced” adolescent and adult rats). This arrangement yielded the following groups of animals: Naïve juveniles (n = 10), naïve adolescents (n = 13), naïve adults (n = 8), experienced adolescents (n = 7), and experienced adults (n = 12).

### Experiment 2: Quantification of Dopamine D1 Receptor Subtype Expression

#### RNA preparation

Separately, 18 non-trained rats were euthanized by decapitation, the brain was quickly removed from the head, and samples of brain regions of interest from both right and left hemispheres were quickly dissected, flash-frozen in liquid nitrogen, and stored at −80°C until RNA extraction. Total RNA was extracted from brain samples from juvenile (n = 6), adolescent (n = 6) and adult (n = 6) rats using TRI Reagent with glass fiber filter purification (RiboPure Kit, Applied Biosystems/Ambion, Austin, TX). After extraction, total RNA pellets were resuspended in RNAsecure and DNAse treated using DNAse-free (both from Applied Biosystems/Ambion). The quality and concentrations of RNA were evaluated using a Take Three application and BioTek Synergy 2 multimodal plate reader (Biotek, Winooski, VT) to measure the ratio of optical density at 260/280 nm wavelengths. Samples were then diluted to 0.1 µg/µL as recommended by Applied Biosystems, Inc. The RNA samples were stored at −80°C until subsequent use. To obtain cDNA from RNA samples a High Capacity RNA-to-cDNA Kit (PN 4387406, Applied Biosystems) was used, the quality and concentrations of cDNA were evaluated using the Take Three application and samples were diluted to identical concentrations.

#### Quantitative real time PCR

Gene expression was evaluated using quantitative real-time polymerase chain reaction (qPCR). Assays for rat GAPDH (NCBI Reference NM017008.3, M29341.1, M17701.1, M11561.1, X02231.1, AB017801.1, BC087743.1 and BC059110.1) and rat D1 dopamine receptor (NCBI Reference NM012546.2 and M35077.1) were obtained commercially (Applied Biosystems). Each assay was conducted as a real-time PCR assay in a 48-well format using TaqMan EZ RT-PCR kits (Applied Biosystems, Foster City, CA; #4331182). Each 20-µl reaction was run in triplicate, and contained 9 µl cDNA diluted in nuclease-free water, 10 µl of TaqMan Universal PCR Master Mix, and 1 µl of 20X TaqMan Gene Expression Assay Mix (containing unlabeled forward and reverse PCR primers; GAPDH primer sequence AGGAGTCCCCATCCCAACTCAGCCC; D1 primer sequence TCTAGAAAAGATCCAACCTGTCACA), and TaqMan MGB probe for the genes of interest, labeled with FAM (probe sequence proprietary to Applied Biosystems; assay ID for GAPDH: Rn01775763g1; assay ID for D1: Rn03062203s1). The thermocycle conditions were: 50°C for 2 min, hold for 10 min at 95°C, followed immediately by 40 cycles of PCR amplification (melt at 95°C for 15 sec, anneal and extend at 60°C for 60 sec) using a StepOne Real-Time PCR system (Applied Biosystems). Controls for primer efficiency were run in the same experiments as our experimental samples, as were controls for the reporter dye and blank (water-only) controls.

#### Quantification

All mRNA levels were normalized to mRNA levels for a ubiquitous reference gene (GAPDH). Prior to selecting GAPDH as the reference gene, we confirmed that the gene was stably expressed during development, and that its abundance showed a strong correlation with the total amount of mRNA present in the samples. Quantification of normalized samples was performed using the Comparative CT method of Livak and Schmittgen [36]. This method relates the PCR signal of the target transcript in a treatment group to that of another sample such as an untreated control. In some samples, the level of D1 receptor mRNA was undetectable, despite an abundance of GAPDH mRNA present. These samples were subjected to further analysis for D1 receptor mRNA only if the GAPDH values were within 1.6 standard deviation of the mean level of GAPDH expression; otherwise, the samples were removed from further analyses. Samples retained for analysis were assigned a cycle value of 45 for D1 receptor mRNA, and all subsequent statistical analyses were carried out using nonparametric statistical tests of median values (Mann-Whitney *U* test) to protect against the possibility of falsely reporting a mean difference between groups because of the arbitrary assignment of cycle value.

### Experiment 3: Immunohistochemical Analysis of Dopamine D1 Receptor Subtype Expression

To assess the distribution of dopamine D1 receptor-expressing cells within OFC, 19 rats were deeply anaesthetized with barbiturate (Euthasol; Virbac Animal Health, Inc., Fort Worth, TX) and transcardially perfused with phosphate buffered saline (PBS; 0.9% NaCl with 0.01 M phosphate buffer at pH 7.4) followed by 4% formaldehyde freshly depolymerized from paraformaldehyde. Brains were then removed from the skull and cryoprotected in 30% sucrose at 4°C. Sections were cut at 40 µm in the coronal plane using a cryostat and were mounted on electrostatically-charged slides. Immunohistochemical localization of dopamine receptors was performed on slides, using a modification of previously described protocols [37], [38]. Briefly, sections were prewashed in phosphate buffered saline (PBS), nonspecific staining reduced with a wash including bovine serum albumin (BSA) and normal goat serum, followed by overnight incubation at 4°C in primary antiserum (1∶200 polyclonal rabbit anti-D1 receptor IgG, Millipore ABN20). The following day, sections were washed in PBS+BSA+Triton X-100, then incubated in biotinylated goat-anti-rabbit secondary antibody for 1 hour, washed in PBS, incubated in Vectastain ABC solution (Vector Laboratories, Inc.; PK-6100) for 1 hour, and reacted with diaminobenzidine and 0.01% H_2_O_2_ for 5 minutes at 25°C. Sections were dried, dehydrated and coverslipped.

Each region of interest (ROI) was examined on sections randomly chosen for quantification. The ROIs included lateral (LO) and ventrolateral orbital (VLO) regions of the OFC, and dorsal (APCd) and ventral (APCv) regions of the anterior piriform cortex. A microscope (Nikon Instruments) connected to a computer running Neurolucida software (MicroBrightField, Inc., Williston, VT) was used to visualize sections and create boxes which were randomly placed overlying ROIs (with alignment adjusted minimally, as necessary, to ensure that the borders of the sampling boxes were within the borders of the ROI), and the number of immunopositive cells within the sampling box was determined. At all times, experimenters were blind to the age-group identity of the tissue being quantified. Counts were made of tissue from juvenile (n = 7), adolescent (n = 6) and adult (n = 6) animals. Statistical analyses were performed on raw cell counts using ANOVA, and a significant F test was followed by post-hoc tests of group means using Tukey’s HSD.

### Experiment 4: Contribution of Dopamine Receptors during Adolescent Learning

Three additional groups of naïve adolescent rats were trained on the digging task (see *Experiment 1* above). One day after rats had successfully learned to dig in unscented sand, they proceeded to the odor-association task. Thirty-minutes before starting the odor-association task, these animals were administered a subcutaneous injection of either 0.33 mg/kg dopamine D1 receptor agonist SKF-38393 (SKF; Sigma-Aldrich; n = 6), 0.125 mg/kg of the dopamine D2 antagonist eticlopride (Sigma-Aldrich; n = 6) or saline (n = 6). As before, animals were tested on the odor-association task and the number of trials to criterion (8 out of 10 consecutive trials correct) was recorded. Animals that required more than one session were injected thirty minutes before each session, but no more frequently than once in a 12 hour period. Data were analyzed using ANOVA and followed up with Dunnet’s test to compare injected group means to the naïve adolescent group mean.

## Results

### Experiment 1: Age-related Differences in Associative Learning

To determine whether rats displayed differences in associative learning during development through adolescence and into adulthood, we trained them on an associative learning task described previously [34]–[35]. We used an odor-guided sand-digging task because rats can learn to dig for palatable reward within a few sessions, and subsequently learn to associate odors with the reward very quickly. The learned association between an odor and the reward (i.e., the predictive value of the odor) has been shown to be encoded by a network which includes the OFC and piriform cortex in adults (see Introduction).

**Figure 1:**
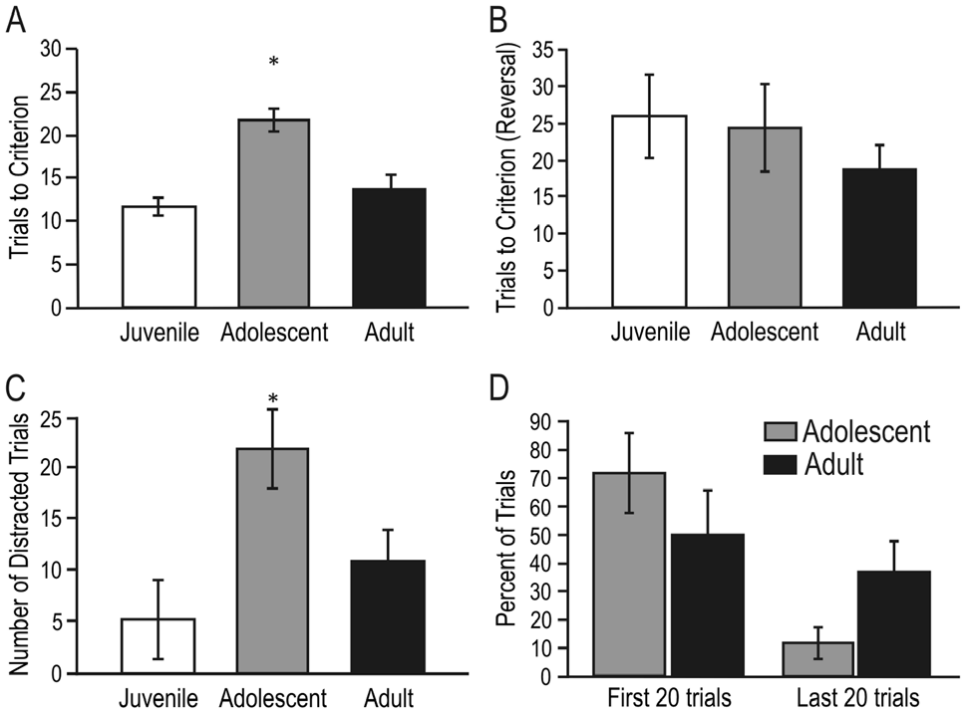
Adolescent rats learn an odor-guided association more slowly than juvenile or adult rats. A) Average number of trials (± SE) to reach criterion (8 out of 10 consecutive trials correct) on an olfactory-guided associative learning task. Adolescent rats required significantly more trials to learn the odor association than either juvenile or adult rats (*, *p*<0.002 and *p*<0.02, respectively) **B)** Average number of trials (± SE) to reach criterion on the reversal task. There were no differences between groups on this task; however, juvenile and adult rats took longer to reach criterion on reversal than on the initial odor association task (compare with panel **A**). **C)** Average number of trials (± SE) that rats displayed “distracted” behavior not directed at the sand-filled cups (e.g., grooming, exploration). These trials were not included in the data shown in panel **A**. While juvenile rats were rarely distracted, adolescent rats displayed significantly more distracted trials than either adult or juvenile animals (*, *p*<0.05 and *p*<0.02, respectively). The average number of distracted trials exhibited by adult rats was not significantly different from juvenile animals. **D)** In adolescent rats, distractibility decreased over the course of the learning task; distracted trials in adolescent rats were significantly more prevalent in the first 20 trials of the odor association task than in the 20 trials immediately prior to criterion. No such change in distractibility was seen in adult animals across the duration of the task.

An ANOVA revealed a significant main effect of age on the number of trials to learn the odor-association task (F_(2,28)_ = 8.27, p<0.002), which was investigated further using Tukey’s HSD post-hoc tests. After learning to dig for palatable reward, juvenile animals naïve to the task were able to learn the predictive value of an odor (8 out of 10 consecutive trials correct) in an average of 11.7 trials (±1.8 SE), which was about the same number of trials as naïve adult rats (mean = 13.5 trials to criterion ±1.9 SE; difference = 1.8; *p*>0.9). In contrast, naïve adolescent animals required a significantly greater number of trials to reach criterion (mean = 21.2 trials ±1.5 SE), than either the naïve juvenile (difference = 9.5; *p*<0.002) or the naïve adult animals (difference = 7.7; *p*<0.02; Figure 1A). Interestingly, when rats were tested on a reversal task (i.e., the odor previously associated with the reward signaled the absence of the reward, and the previously unrewarded odor signaled its presence), there were no significant differences in trials to criterion; reversal learning took juvenile and adult animals approximately twice as many trials to learn as the odor association task, while adolescent animals learned this task at the same rate as the initial pairing (Figure 1B).

Only “active” trials where the rats were engaged in the digging task were used to determine criterion for learning, so the slower learning exhibited by adolescents for the initial odor pairing could not be attributed to an increase in incompatible behavior (such as grooming or investigation). However, a separate analysis of these “distracted” trials (i.e, trials during which the rat’s behavior was not directed toward the digging task) revealed a significant effect of age on the number of distracted trials exhibited in the odor association task (ANOVA F_(6, 45)_ = 3.399; *p*<0.01). Post-hoc tests with Tukey’s HSD revealed that adolescent animals displayed significantly more distracted behavior than either juveniles or adults (Figure 1C) during the odor association task (there were no differences among groups in the number of distracted trials during reversal).; adolescents spent significantly more time engaged in behavior that was not task-related (mean = 22.8 trials ±4.0 SE) than juvenile animals, where distracted behavior was all but absent (mean = 5.0 trials ±4.5 SE; difference = 17.8, *p*<0.02). Adult animals displayed an intermediate level of distracted behavior (10.4 trials ±5.0 SE) that was significantly lower than that displayed by adolescent animals (*p*<0.05), but not significantly different than juvenile animals.

Interestingly, distractibility decreased in adolescent rats as they learned the task (Figure 1D); during the first 20 trials of the odor association, adolescent rats displayed an average of 14 (±2.88 SE) distracted trials (or 70.8% of trials), while the 20 trials immediately preceding criterion contained an average of just 2.3 distracted trials (±1.0 SE; 11.7%). This pattern was not seen in adult animals, which displayed an average of 10 (±3.3 SE) distracted trials over the first 20 odor association trials (50%), and an average of 7.5 (±2.2 SE; 37.5%;) distracted trials over the last 20 trials before criterion was reached.

**Figure 2:**
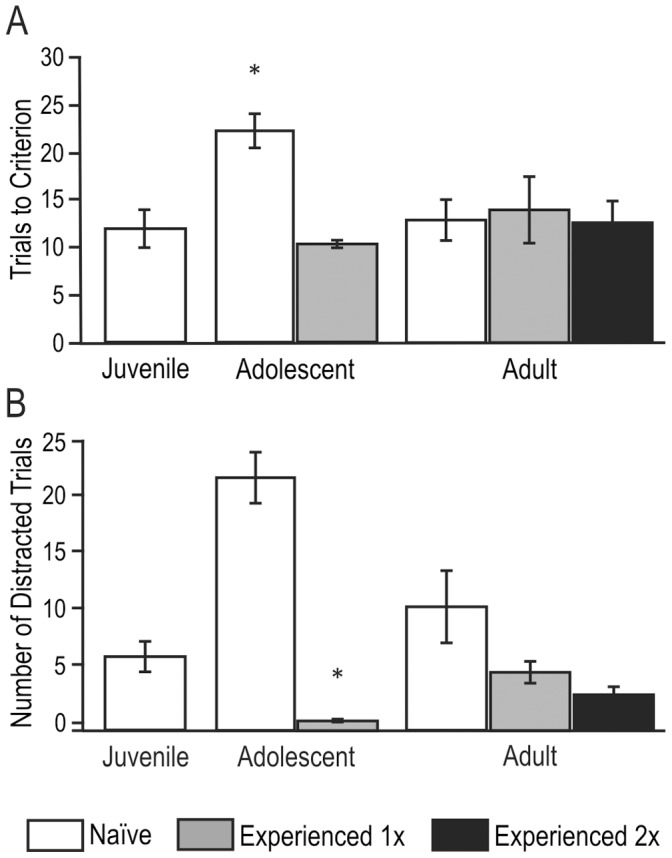
Effects of previous experience. A) Average number of trials (± SE) to reach criterion on an olfactory-guided associative learning task for animals that were naïve to the task (white bars), and for animals that had previously experienced the associative learning task once (gray bars) or twice (black bars). While naïve adolescent rats required significantly more trials to learn the odor association than either juvenile or adult rats, experience with the task as a juvenile significantly reduced the number of trials required to reach criterion in adolescent animals (*p<0.005 compared to naïve adolescents). There were no significant differences between experienced adolescent animals and juveniles or any adult groups. **B)** Average number of trials (± SE) that rats displayed “distracted” behavior. Experience with the task significantly reduced the number of distracted trials displayed by adolescent rats (*p<0.01 compared to naïve adolescents). There was no apparent effect of prior task experience on distractibility among groups of adult rats.

Additionally, we found that when juvenile animals that performed the odor association task were tested on a new odor association as an adolescent, their learning improved (ANOVA F_(4,40)_ = 5.56, *p*<0.02). The experienced adolescent animals (which had learned the association task with different odors as juveniles) required significantly fewer trials to meet criteria than naïve adolescents (mean = 10.25±0.25 SE; difference = 10.95, *p*<0.005 with Tukey’s HSD; Figure 2A). In addition, juvenile experience significantly reduced the number of distracted trials displayed in adolescence (*p*<0.01; Figure 2B) to levels comparable to that of juveniles. No significant differences were found between naïve and experienced adult animals.

### Experiment 2: Quantitative Real-time PCR of D1 Receptor

**Figure 3:**
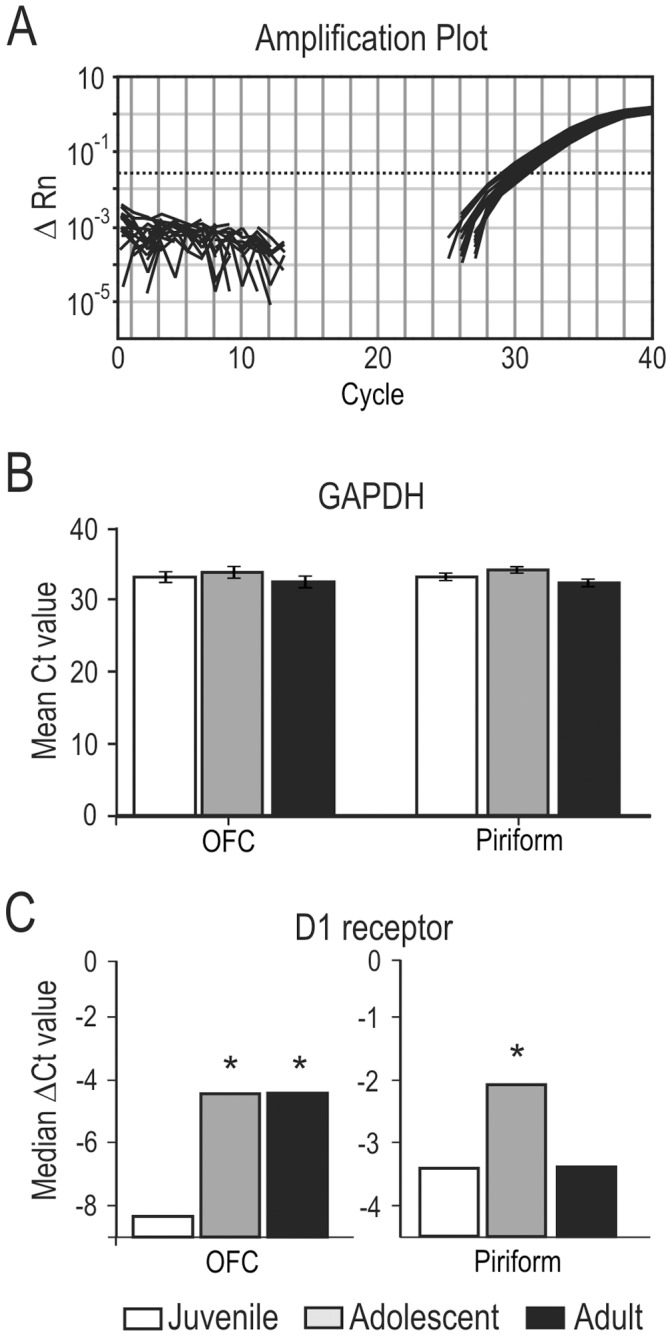
Dopamine D1 receptor mRNA expression increases in adolescence. A) Real-time quantitative PCR (qPCR) amplification plot showing levels of mRNA for GAPDH, a ubiquitous reference gene. Amplification plots for thirty-two samples are shown (one line for each sample), taken from both OFC and piriform cortex at different ages. The logarithmic scale ΔRn is the magnitude of the fluorescence signal from the reporter dye normalized to the fluorescence of the passive reference signal, with the normalized baseline fluorescence subtracted. The dotted line displays the calculated threshold value within the exponential growth phase of the PCR that defined the threshold cycle (Ct) for each sample. The threshold value for this amplification plot was 0.027. **B)** Average Ct (± SE) for GAPDH by age. Note that GAPDH is expressed at stable levels throughout postnatal development in both the OFC and piriform cortex. Averages are for a minimum of eight samples per age group, run in triplicate. Samples that were run as a control (either without reverse transcriptase or without the target primers and probes) showed no amplification (data not shown). Juveniles are designated by white bars, adolescents by gray bars, and adults by black bars. **C)** Average ΔCt levels for D1 receptor mRNA relative to GAPDH levels as a function of age. Unlike GAPDH, D1 receptor mRNA levels are developmentally regulated in both OFC and piriform cortex. Results are inverted for display so that lower values denote lower levels of mRNA. In both OFC and piriform cortex, mRNA levels were significantly higher in adolescent rats (gray bars) compared to juveniles (white bars; *p<0.01). In the OFC of adult rats (black bars), these values remained significantly higher than juvenile levels. In piriform cortex, adult mRNA levels were not significantly different from juvenile rats.

Levels of mRNA for the D1 receptor were determined for OFC and piriform cortex from both hemispheres of juvenile, adolescent and adult rats, normalized to mRNA levels for a ubiquitous reference gene (GAPDH). Prior to selecting GAPDH as the reference gene, we confirmed that it was stably expressed during postnatal development, and that its abundance showed a strong correlation with the total amount of mRNA present in the samples. Indeed, no differences in mean threshold cycle (Ct) values for GAPDH expression were observed among age groups for either the OFC or piriform cortex (Figure 3).

The expression of D1 receptor mRNA in OFC and piriform cortex showed a clear developmental regulation in rats. As illustrated in Figure 3, both adolescent and adult animals had significantly elevated levels of D1 receptor mRNA expression in the OFC compared to juvenile animals (21–23 d; juvenile vs. adolescent: *U_(9, 10)_* = 78, *p*<0.01; juvenile vs. adult, *U_(9, 10)_* = 52, *p*<0.01). Similarly, adolescent rats displayed significantly higher levels of D1 receptor mRNA in piriform cortex compared to juvenile and adult levels (*U_(21,11)_* = 91, *p*<0.01).

### Experiment 3: Immunohistochemical Detection of D1 Receptor Protein Expression

**Figure 4:**
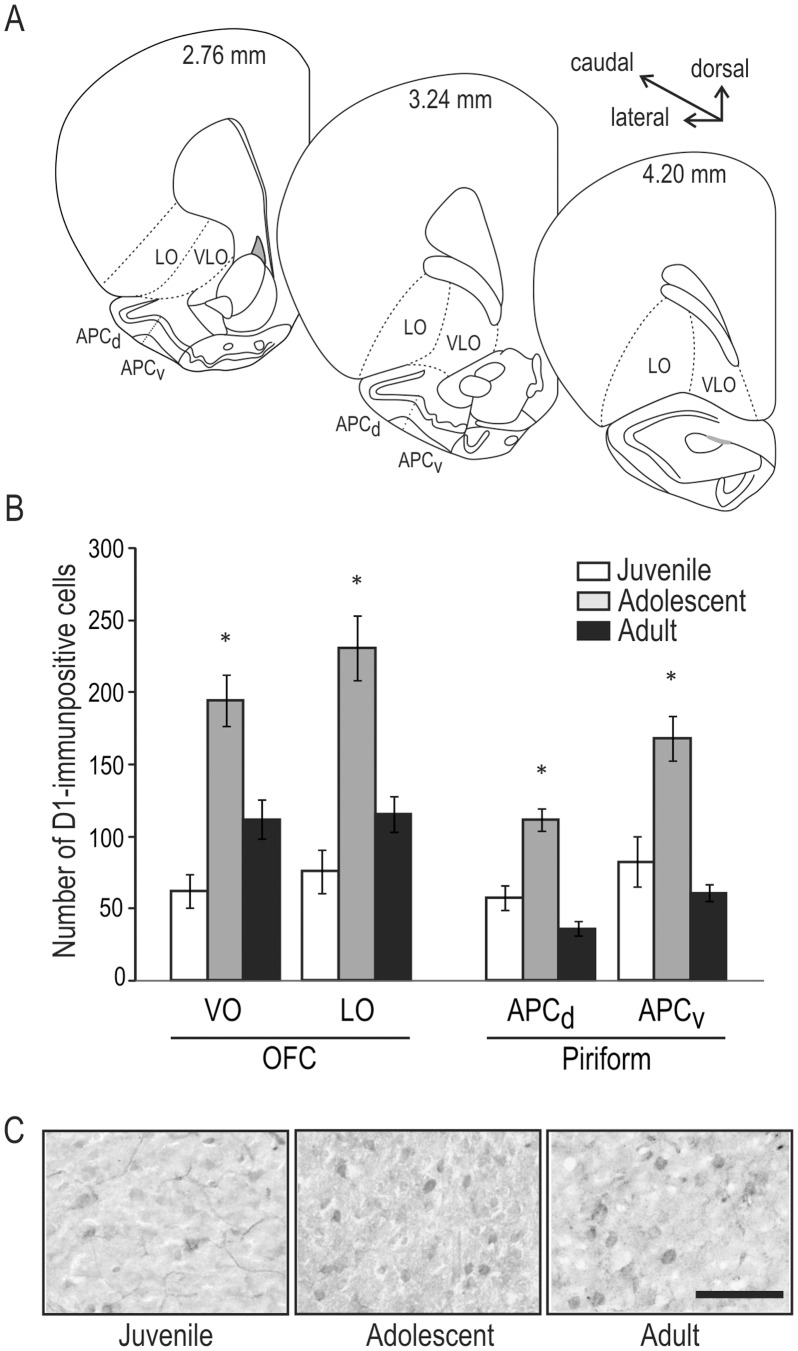
Dopamine D1 receptor protein expression increases in adolescence. A) Subregions of the OFC (LO and VLO) and piriform cortex (APCd and APCv) sampled to determine the number of D1 receptor-immunopositive cells at each age. Drawings of the right hemisphere shown, adapted from Paxinos and Watson (2007). Uniform sampling boxes were placed randomly within each area and all immunopositive cells were counted. All counts were performed by experimenters blind to the age and identity of the sections. **B)** Average number of D1-immunopositive cells counted in each sampled region for juvenile (white bars), adolescent (gray bars) and adult rats (black bars). Within each subregion, a significant increase in the number of immunolabeled cells was found in adolescence compared with either juveniles or adults (*p<0.001). **C)** High-power photomicrographs of sampled regions in the OFC of juvenile, adolescent and adult rats. Scale bar = 100 µm, applies to all photomicrographs in C.

Finding differences in D1 receptor mRNA levels among age groups within the OFC and piriform cortex prompted an examination of D1 receptor protein expression in these areas. In particular, we were interested in whether the increase in D1 receptor mRNA was reflected in either the number of D1 receptor-expressing cells or their distribution within these areas. Quantification of D1 receptor-expressing cells was performed by counting all immunopositive cells within randomly selected regions of OFC and piriform cortex (see Materials and Methods for details; Figure 4A). In both cortical areas, we found an overall main effect of age (ANOVA; F_(14, 435)_ = 14.766, *p*<0.0001; Figure 4B). Post-hoc analyses with Tukey’s HSD revealed that adolescent rats displayed a significantly higher number of D1-immunolabeled cells in each region examined than either juvenile or adult animals (*p*<0.001 for each comparison). Despite these large changes in the overall number of cells in each cortical area during adolescence, there were no apparent changes in the overall distribution of cells expressing the D1 receptor; cells appeared distributed throughout the regions examined, with no evidence for clustering (Figure 4C).

### Experiment 4: Contribution of Dopamine Receptors to Adolescent Learning

**Figure 5:**
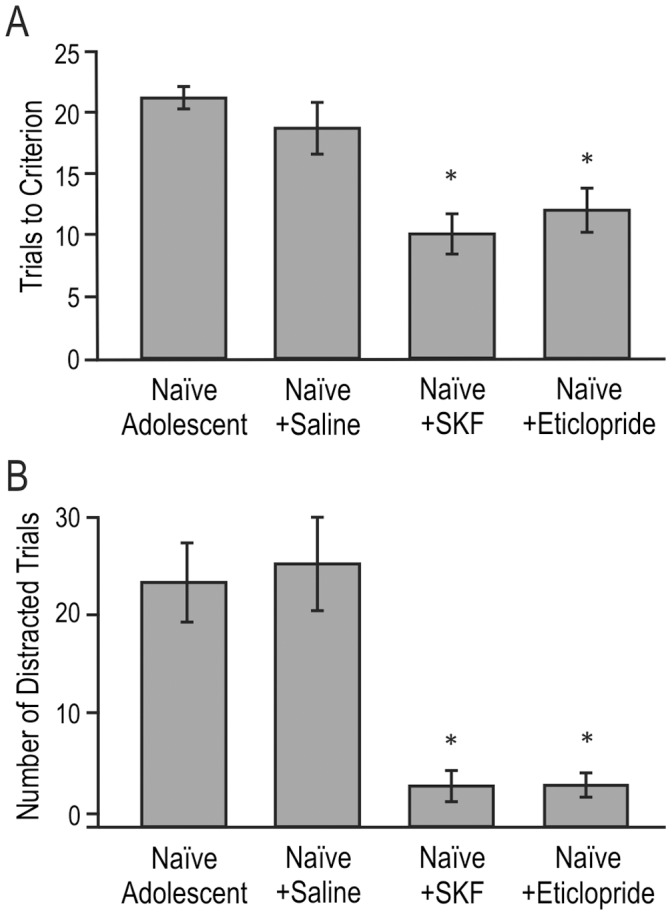
Drugs affecting dopamine receptors improve associative task performance and reduce distracted trials in adolescent rats. A) Average number of trials (± SE) to reach criterion for naïve and drug-injected adolescent animals. Both SKF and eticlopride (but not saline) reduced the number of trials required for animals to learn the odor association (*p<0.01 compared to naïve adolescents). **B)** Average number of trials (± SE) on which adolescent rats displayed “distracted” behavior. Both SKF and eticlopride (but not saline) significantly decreased the number of distracted trials exhibited by adolescent rats during the odor association task compared to naïve adolescents (*p<0.05).

To examine whether the changes in D1 receptor levels contributed to the differences in odor-association task performance, we trained three additional groups of naïve adolescent animals that were treated with drugs targeting dopamine receptors. Thirty minutes prior to the odor association session, these animals received a subcutaneous injection of a low dose of either the D1 agonist SKF (0.33 mg/kg), or the D2 antagonist eticlopride (0.0125 mg/kg). Control animals were injected with an equivalent volume of saline. The animals were then tested on the odor association task, and the number of trials to criterion was recorded. An ANOVA revealed a significant effect of the injection (F_(3, 24)_ = 14.59; *p*<0.001). Post-hoc analyses revealed that naïve saline-injected adolescents took as long to learn (mean = 18.8 trials to criterion ±1.6 SE) as uninjected naïve adolescents (21.2 trials ±1.2 SE; difference = 2.4, *p*>0.5; Figure 5A). In contrast, all naïve adolescent animals that received a low dose of SKF learned the odor association in a single session (mean = 10.0 trials ±1.4 SE), requiring significantly fewer trials to learn than either naïve adolescents (difference = 11.2 trials, *p*<0.001) or saline-injected controls (difference = 8.8 trials, *p*<0.03; Figure 5A). Adolescent animals injected with eticlopride achieved similar results, learning the odor association in significantly fewer trials than naïve adolescent animals (mean = 12.2±2.3 trials; difference = 9.0, *p*<0.01; Figure 5A). The effects of low-dose SKF and eticlopride on learning the odor association was comparable to the effect of juvenile experience; there were no significant differences between injected animals and adolescents that had learned odor discrimination as a juvenile. As before, there were no significant differences among groups for the reversal task.

Subcutaneous injection of drugs also reduced the number of distracted trials displayed by adolescent rats (Figure 5B). While saline injection had no effect on the number of distracted trials displayed (mean = 25.8 trials ±8.1 SE; *p*>0.20), injection of SKF significantly decreased the number of distracted trials (mean = 2.25±1.4 SE) compared with naïve rats (mean = 22.8 trials ±6.1 SE; *p*<0.05). Similarly, eticlopride reduced the number of distracted trials to a mean of 2.2 trials (±1.4 SE), significantly lower than naïve adolescent rats (*p*<0.05).

## Discussion

### Postnatal Development of Associative Learning Mirrors Dopaminergic Development

The OFC and piriform cortex, along with associated subcortical structures, play a role in encoding the predictive value of cues during odor-guided associative tasks [1], [2], [39]–[41]. We have demonstrated that adolescent rats require significantly more trials to learn an odor-guided associative learning task than either juvenile or adult animals. This learning deficit is exacerbated by–but does not result from–an increase in the number of “distracted” trials exhibited by adolescent rats. We also have found that D1 receptor expression in the OFC and piriform cortex increases significantly during adolescence, with increases both in the mRNA levels and in the number of D1 receptor immunolabeled cells compared with juveniles. This postnatal development parallels that of dopamine receptors in other frontal cortical regions (see [32] for a review), and peak levels of dopamine receptor mRNA and protein expression coincide with the maturation of dopaminergic projections in the OFC and other frontal cortices [29]. Together, these results suggest that maturation of frontal dopamine circuitry may be important for associative learning as rats transition from adolescence to adulthood.

The deficit in learning we observed in adolescence could be ameliorated by a single, low-dose, systemic injection of a drug that increased postsynaptic dopamine receptor activation (either the D1 receptor agonist SKF-38393 or the D2 receptor antagonist eticlopride), but the systemic injections employed in this study preclude localizing the site of action of these drugs. In particular, the olfactory bulb is rich with dopaminergic cells, and dopamine has been shown to suppress the input to the olfactory bulb from the olfactory nerve [42]–[44]. Because this effect is mediated solely by the D2 receptor [43], it may be argued that in our study, eticlopride acted by modulating olfactory processing rather than associative learning *per se*. However, releasing suppression of the olfactory nerve input would be expected to degrade associative-cue odor signals relative to background odors [43], resulting in impaired performance rather than the improved performance we found in adolescent animals in our study. Similarly, although D1 receptors are widespread in the brain, their expression is extremely low in the olfactory bulb [15], [45], so the observed improvement in task performance in SKF-injected animals could not be attributed to an influence on olfactory sensory processing in the olfactory bulb. Thus, although our drug injections certainly had effects throughout the brain, the improvement in task performance resulting from these injections are more likely to have resulted from their effects on central networks underlying associative learning than from their effects on processing of olfactory cues. Previous work demonstrating that drugs targeting dopamine receptors reduce impulsive behavior and improve learning in both rats (e.g., [46]–[47]) and humans (e.g., [48]) lends further support to the notion that age-associated differences in learning are mediated at least in part by differences in levels of dopamine receptor activation.

The changes in D1 receptor expression and distribution we observed in the OFC and piriform cortex are some of many ongoing modifications in cortical circuitry during adolescence. Cortical structures important for associative learning in the adult rat are immature at birth, and significant changes in the morphology and physiology of structures involved with olfactory-guided behavior occur during the first four weeks in the rat [49]–[56]. Despite this fact, associative learning and experience-dependent plasticity can be exhibited at very young ages (e.g., [52], [57]). Therefore, associative learning in neonatal and juvenile rats may differ from that in adult rats, perhaps relying on a separate learning network or set of brain structures. If so, a transition to adult-like learning may occur during adolescence, which may result in a period of decreased performance on associative learning tasks. Interestingly, experience with the odor association task as a juvenile in our study decreased both the number of trials required to reach learning criterion and the number of distracted trials in adolescent rats, suggesting that such a transition–if it occurs–may be improved or accelerated by experience.

### Behavioral Implications and Adaptive Significance

During adolescence, rats begin to leave the nest [58]–[59], and during this time they display more exploratory behavior in novel environments than adult rats, display more impulsive and distracted behavior, and learn odor associations more slowly (Figures 1–2 and 5; [60]). Each of these behaviors may be influenced by activity in the OFC [1], [5], [47], [61]–[62]. Interestingly, in situations where D1 receptors are more highly activated, adolescent rats display decreased distractibility and exhibit learning as well as adults (Figure 5; see also [56], [63]–[64]).

What could be the survival advantage of increased distractibility and slower learning during adolescence? Perhaps learning only those associations which are repeatedly confirmed or which are highly rewarding leads to greater behavioral variety, supporting more exploration and risk-taking and driving adolescent animals to leave the nest. In this way, behaviors that are important for survival such as foraging, colonizing new territory, and engaging in social behaviors are promoted. In situations where D1 receptors are more highly activated, either by a highly rewarding situation or through external manipulation (i.e. drugs), behavior becomes more focused and associations learned more rapidly.
